# Marine Low Molecular Weight Natural Products as Potential Cancer Preventive Compounds

**DOI:** 10.3390/md12020636

**Published:** 2014-01-27

**Authors:** Valentin A. Stonik, Sergey N. Fedorov

**Affiliations:** 1 G.B. Elyakov Pacific Institute of Bioorganic Chemistry, Far-Eastern Branch of the Russian Academy of Science, Prospect 100 let Vladivostoku, 159, Vladivostok 690950, Russia; E-Mail: fedorov@piboc.dvo.ru; 2 School of Natural Sciences, Far East Federal University, Vladivostok 690950, Russia

**Keywords:** marine organisms, secondary metabolites, cancer preventive, chemopreventive, anticarcinogenic, mechanisms of action

## Abstract

Due to taxonomic positions and special living environments, marine organisms produce secondary metabolites that possess unique structures and biological activities. This review is devoted to recently isolated and/or earlier described marine compounds with potential or established cancer preventive activities, their biological sources, molecular mechanisms of their action, and their associations with human health and nutrition. The review covers literature published in 2003–2013 years and focuses on findings of the last 2 years.

## 1. Introduction

The outstanding biological/biochemical diversity of marine environment serves as a source of inspiration and attracts great interest of chemists and pharmacologists. Some new secondary metabolites of marine origin, produced by diverse marine organisms are important leads for the development of anti-cancer pharmaceuticals. Recent research has also included previously known physiologically active marine natural products, which themselves or their synthetic derivatives are applicable in cancer therapy or cancer prevention [1,2,3,4,5,6,7,8]. 

The latter involves the employment of natural or synthetic compounds for prevention, suppression or reversion of the process of carcinogenesis [9]. This area is a fast growing scientific field, because cancer prevention is suggested to be a promising way to decrease the number of human cancer cases. Increasing attention is also being paid to the possibility of application of natural products with cancer preventive properties for individuals at the high risk of neoplastic development [10]. 

The carcinogenic process consists of three phases: initiation, promotion, and progression [11,12]. Initiation and progression are relatively fulminant and irreversible, so promotion is the most appropriate phase for chemoprevention [13,14]. Anti-promotional agents selectively inhibit the process of preneoplastic changes. It often requires the continuous presence of chemopreventive agents [15]. Showing, as a rule, low cytotoxic activities against tumor cells, cancer preventive compounds inhibit the malignant transformation of normal cells into tumor cells, or influence other stages of carcinogenesis [15,16,17,18]. Some of them are known antagonists of cancer promoters and oncogenes, others stimulate anticancer immunity, induce apoptosis, or arrest the cell cycle, inhibit tumor invasion, angiogenesis, or inflammation [19,20,21,22]. 

Recent progress in the research of marine anticancer compounds resulted in identification of various molecular targets for chemopreventive drugs, such as indoleamine 2,3-dioxygenase, histone deacetylases and methyl-transferases, matrix metalloproteinases (MMPs), hypoxia-inducible factor-1 (HIF-1), retinoic acid receptor, peroxisome proliferator-activated receptor (PPAR) isoforms, ubiquitin-proteasome pathway, oncogenic nuclear factors activator protein-1 (AP-1) and nuclear factor-κB (NF-κB), tumor suppressor protein p53-mouse double minute 2 protein (Mdm2) interaction, different tumor-related kinases, *etc.* [23,24,25,26,27,28,29,30,31,32]. 

Natural products are particularly useful as cancer preventive or anticarcinogenic agents if they show good availability, low toxicity, suitability for oral application, and a vast variety of mechanisms of their action. It is believed more and more that cancer may be prevented or at least delayed and inhibited through the use of natural compounds [20]. In fact, there are clear links between human cancer and diet, and seafood is considered to be exclusively useful with respect to cancer prevention.

Herein, we review the studies, mainly published in recent years, on several groups of the marine naturally occurring compounds, which are potentially useful for cancer prevention as can be judged from *in vitro* and/or *in vivo* results. Mostly, these compounds are noncytotoxic, or at least show their anticancer properties at nontoxic concentrations. Our review highlights biological sources, structures and mechanisms of action of the marine lipids, carotenoids, glycosides, terpenoids, alkaloids, and other marine natural products that are currently undergoing evaluation as cancer preventive agents either in laboratories or in clinical trials.

## 2. Marine Lipids

**Figure 1:**
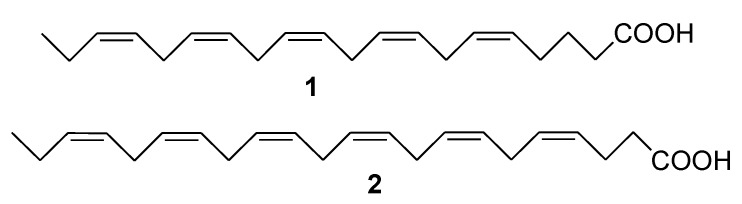
Structures of the compounds **1** and **2**.

Many marine edible organisms contain lipids enriched by polyunsaturated fatty acids (PUFAs). Marine ω-3 fatty acids, mainly consisting of eicosapentaenoic (EPA) (**1**, Figure 1) and docosahexaenoic acids (DHA) (**2**, Figure 1), compete in various enzymatic processes with ω-6 polyunsaturated acids such as arachidonic acid. The role played by ω-3 (DHA and EPA) and the ratio of ω-3/ω-6 PUFAs needed to optimally suppress the development of most cancers, including breast, colon, prostate, liver, and pancreatic tumors, were established in many experimental studies [33,34,35]. The mechanisms by which ω-3 PUFAs are thought to possess antineoplastic activity, as well as preclinical and current clinical trials, investigating the potential therapeutic roles of ω-3 PUFAs at different stages of colorectal carcinogenesis, have been reported [36].

Recently, in a large colonoscopy-based case-control study that involved 5307 Western individuals, the association of dietary PUFA intake and the risk of colorectal polyps were evaluated. It was found that the dietary intake of the marine-derived ω-3 PUFAs was associated with a decreased risk of adenomatous polyps in women, but not in men. For women, higher intake of the marine-derived ω-3 PUFAs was associated with lower levels of prostaglandin E2, which may suggest that the alteration of eicosanoid production is an important mechanism that underlies the chemopreventive effects of the marine- derived ω-3 PUFAs [37]. 

Another recent study showed that marine ω-3 PUFA ameliorated inflammation, fibrosis, and vascular abnormalities in fat tissue through a decrease in adipose tissue macrophages, an increase in adipose capillaries, and a decrease in macrophage chemoattractant protein 1 (MCP-1) levels [38]. 

Numerous experiments on animals confirmed the cancer preventive properties of fish oils and ω-3 fatty acids from the marine sources. The chemopreventive effect of *ω*3 PUFA was evaluated in rats with colorectal cancer, induced via the carcinogen 1,2-dimethylhydrazine (DMH). Lower levels of aberrant crypt foci were found in rats fed with *ω*3 PUFA. In addition, this group of carcinogen-treated rats showed greater expression of transforming growth factor *β* and lower interleukin-8 expression, resulting in a protective effect on the colonic precancerous mucosa and a beneficial effect on inflammatory modulation [39]. Using the Fat-1 mice, a genetic model that synthesizes long-chain ω-3 PUFAs *de novo*, it was shown that ω-3 PUFAs can modulate the colonic mucosal microenvironment to suppress Th17 cell accumulation and inflammatory damage following the induction of chronic colitis [40]. The anti-inflammatory properties of ω-6 docosapentaenoic acid derivatives *in vitro* and *in vivo* were demonstrated using murine macrophage RAW 264.7 cells and mice with dextran sodium sulfate (DSS)-induced colitis. Intake of the compounds modulated macrophage function and alleviated the experimental colitis [41]. The regulation of the cellular anti-apoptotic glucose related protein of 78 kDa (GRP78) expression and location have been demonstrated to be a possible route through which DHA can exert pro-apoptotic and antitumoral effects in colon cancer cells [42].

**Figure 2:**
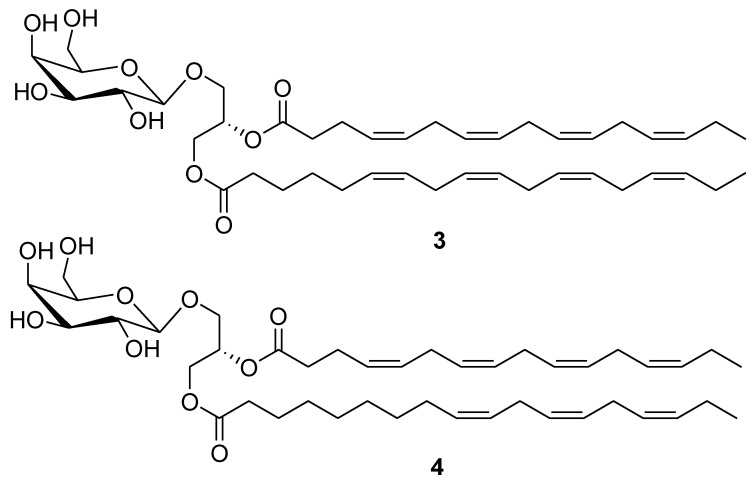
Structures of the compounds **3** and **4**.

Some other marine lipids also showed potential cancer preventive properties. Monogalactosyldiacylglycerols (MGDGs) **3** and **4** (Figure 2) isolated from the marine microalgae *Tetraselmis chui* were tested for their nitric oxide (NO) inhibitory activity on lipopolysaccharide-induced NO production in RAW264.7 macrophage cells. The compounds showed strong NO inhibitory activity compared to NG-methyl-l-arginine acetate salt, a well known NO inhibitor used as a positive control. Isolated MGDGs suppressed NO production through down-regulation of inducible NO synthase protein [43]. 

**Figure 3:**
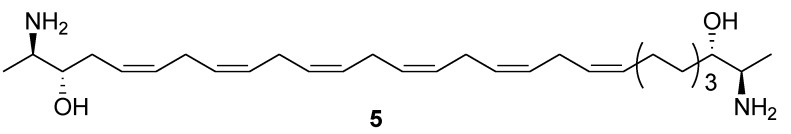
Structure of the compound **5**.

Leucettamol A (**5**, Figure 3), a bipolar lipid that inhibits the formation of the complex composed of the ubiquitin-conjugated E2 enzyme (Ubc13) and ubiquitin-conjugated enzyme variant 1A (Uev1A), was isolated from the marine sponge *Leucetta* aff. *microrhaphis*. Its inhibition of Ubc13-Uev1A interaction was tested by the enzyme-linked immunosorbent assay (ELISA), revealing IC_50_ value of 50 μg/mL. Such inhibitors are presumed to be leads for anti-cancer agents that upregulate activity of the tumor suppressor p53 protein [44]. They may therefore be interesting as the cancer preventive agents.

**Figure 4:**
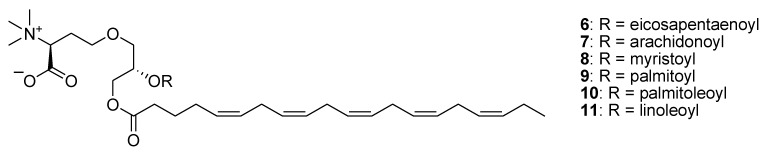
Structures of the compounds **6**–**11**.

Chemical investigation of polar lipids from the marine eustigmatophyte microalga *Nannochloropsis granulata* led to the isolation of six betaine lipid diacylglyceryltrimethylhomoserines (**6**–**11**, Figure 4). The isolated betaine lipids showed dose-dependent nitric oxide (NO) inhibitory activity against lipopolysaccharide-induced nitric oxide production in RAW264.7 macrophage cells. Further study suggested that this activity is exerted by the compounds through downregulation of inducible nitric oxide synthase expression, indicating a possible value as anti-inflammatory agents [45].

## 3. Marine Carotenoids

**Figure 5:**
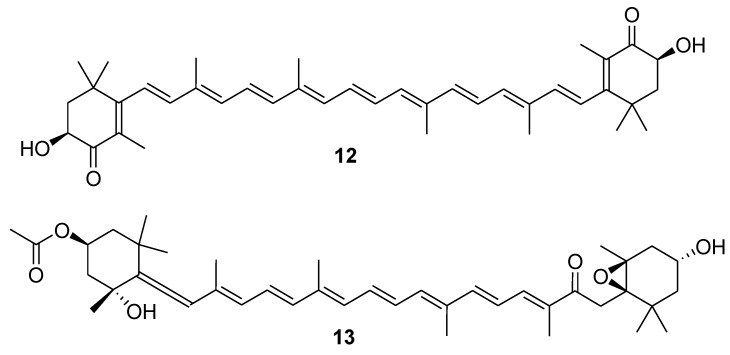
Structures of the compounds **12** and **13**.

Marine carotenoids are fat-soluble pigments that provide bright coloration to animals and seaweeds. The most common marine carotenoids are: Astaxanthin (**12**, Figure 5), fucoxanthin (**13**, Figure 5), canthaxanthin and related carotenoids (xanthophylls) from salmon, shrimp, mollusks, β-carotene from microalgae and some other marine organisms. All these carotenoids showed anticarcinogenic activities *in vitro* and *in vivo* by interrupting several stages of carcinogenesis including initiation, promotion, progression, and metastasis [46,47,48,49,50,51,52,53]. Astaxanthin demonstrated strong antioxidant properties in the variety of *in vitro* and *in vivo* studies and has a great potential for reducing the burden of human diseases related to oxidative damage. Being the effective radical quencher [54], astaxanthin forms radical cations, which are converted into the stable compound very easily *via* electron transfer from vitamin E [55]. Peroxynitrite (ONOO^-^), the reactive nitrogen species, was found to induce various forms of cell oxidative damage such as low-density lipoprotein oxidation, lipid peroxidation, deoxyribonucleic acid (DNA) strand breakage, and nitration of protein tyrosine residues that may lead to tumor-promotion [56]. It was reported that astaxanthin and lutein, a major carotenoid in green algae, could take up peroxynitrite through the formation of 15-nitroastaxanthin and 15-nitrolutein, respectively, thus inhibiting oxidative damage to cells, eventually leading to carcinogenesis [57]. The antioxidant activities of fucoxanthin and its two metabolites, fucoxanthinol and halocynthiaxanthin, were assessed *in vitro* with respect to radical scavenging and singlet oxygen quenching abilities. The 1,1-diphenyl-2-picrylhydrazyl radical scavenging activity of fucoxanthin and fucoxanthinol was higher than that of halocynthiaxanthin, with the effective concentration for 50% scavenging (EC_50_) being 164, 153, and 826 μM, respectively. Hydroxyl radical scavenging activity as measured by the chemiluminescence technique showed that the scavenging activity of fucoxanthin was 7.9 times higher than that of fucoxanthinol and 16.3 times higher than that of halocynthiaxanthin [58]. Fucoxanthin protection against oxidative stress induced by ultraviolet B (UVB) radiation in human fibroblasts might be applicable in the cosmetic industry [59]. The role of the both major marine carotenoids as dietary antioxidants has been suggested to be one of the main mechanisms underlying their preventive effect against cancer and inflammatory diseases. In addition, it has been demonstrated that fucoxanthin improves insulin resistance and decreases blood glucose level, at least in part, through the downregulation of tumor necrosis factor-α (TNF-α) in white adipose tissue (WAT) of animals [60]. 

All xanthophylls studied so far are active as inhibitors of cancer cell proliferation *in vitro* and *in vivo*. The inhibitory effect of astaxanthin against inflammation-related mouse colon carcinogenesis and dextran sulfate sodium (DSS)-induced colitis in male imprinting control region (ICR) mice was investigated. Dietary astaxanthin suppressed colitis and colitis-related colon carcinogenesis, partly through inhibition of NF-κB, and downregulation of the messenger ribonucleic acid (mRNA) expression of inflammatory cytokines, including interleukin-1β (IL-1β), interleukin-6 (IL)-6, and cyclooxygenase (COX)-2 [61]. Astaxanthin from the alga *Haematococcus pluvialis* inhibited growth of HCT-116 and HT-29 human colon cancer cells in a dose- and time-dependent manner by arresting cell cycle progression and promoting apoptosis [62]. Fucoxanthin induced apoptosis and cell cycle arrest in human leukemia HL-60, gastric adenocarcinoma MGC-803, LNCap prostate cancer cells, and primary effusion lymphomas [63,64,65,66]. Another marine carotenoid, siphonaxanthin from the green algae *Codium fragile* potently induced apoptosis in HL-60 cells. The effective apoptotic activity of siphonaxanthin was observed by increased numbers of terminal deoxynucleotidyl transferase dUTP nick end labeling (TUNEL)-positive cells, and by increased chromatin condensation. This induction of apoptosis was associated with a decreased expression of B-cell lymphoma-2 (Bcl-2) protein and a subsequently increased activation of cysteine-aspartic acid protease-3 (caspase-3) [67].

All the properties of astaxanthin and the related marine carotenoids confirm the potential role for these dietary constituents in cancer prevention or inhibition of carcinogenesis and make astaxanthin and fucoxanthin candidates for further investigation as anticancer agents in humans. 

## 4. Glycosides

Various marine invertebrates such as echinoderms, octocorals and sponges contain steroid and triterpene glycosides. These compounds demonstrate a wide spectrum of biological activities including antitumor and cancer preventive effects both *in vitro* and *in vivo* [68,69,70]. All the studied sea cucumbers, including edible species, contain physiologically active triterpene glycosides. These substances are ingested as a part of traditional seafood diet all over the world, especially in Asian countries. Although triterpene glycosides are cytotoxic *in vitro*, uptake via the peroral application reduces their toxicity and induces a rather stimulatory action, mainly on the immune system. Immunostimulatory effects of these natural products are observed when very small noncytotoxic doses of the glycosides are used in *in vitro* and *in vivo* experiments. That is why these compounds are considered to be both anticancer and cancer preventive agents, depending on both the dose and the route of administration. Glycosides from distinctive biological sources differ from each other in their structures and activities, although some similarity in their action has also been indicated [69,70,71]. 

**Figure 6:**
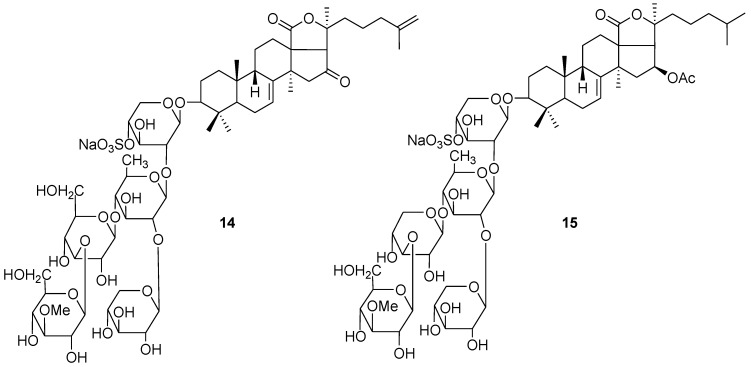
Structures of the compounds **14** and **15**.

The patented pharmaceutical lead, named cumaside, was created on the basis of monosulfated cucumariosides from the edible sea cucumber *Cucumaria japonica*. This preparation demonstrates potent immunostimulatory properties and activates the cellular immunity, in particular lysosomal, phagocytic and bactericide activities of macrophages. Being less toxic than glycosides themselves, it retains immunostimulatory activity and showed inhibition of Ehrlich carcinoma cells *in vivo* [72,73]. When cumaside was injected intraperitoneally in mice at a dose of 200 ng/mouse (days—4 and —1 before tumor inoculation), about 40% of animals were without tumor on day 15 after inoculation. Prophylactic treatment with cumaside (using peroral administration) and subsequent application of 5-fluorouracil suppressed tumor growth by 43% [74]. Recently, it was shown that cucumarioside A_2_-2 (**14**, Figure 6), one of the main components of cumaside, exhibited a cytostatic effect against Ehrlich carcinoma cells at a subcytotoxic range of concentrations by blocking cell proliferation and DNA biosynthesis in the S phase. It also induced apoptosis in tumor cells in a caspase-dependent way, by-passing the activation of the p53-dependent pathway [75].

Cucumariosides I2, B2, and A5 isolated from the sea cucumber *Eupentacta fraudatrix* (Djakonov et Baranova) stimulated an increase of the lysosomal activity of mouse peritoneal macrophages of 15%–16% at doses of 1–5 μg/mL. It was demonstrated that this immunostimulatory activity depended on structures of both the aglycone and carbohydrate chains and was not directly correlated to cytotoxic activities of the glycosides [76]. The structures, antitumor and cytotoxic activities against mouse Ehrlich carcinoma cells and mouse splenic lymphocytes, along with hemolytic activities against mouse erythrocytes, and antifungal activities of twenty eight new triterpene glycosides from three species of holothurians, namely *Eupentacta fraudatrix*, *Cladolabes schmeltzii*, and *Actinocucumis typica1*, were recently investigated. The structure-activity relationships of these compounds were also studied [77,78,79,80,81,82].

As it was recently demonstrated, frondoside А (**15**, Figure 6) from the sea cucumber *Cucumaria okhotensis* and cucumarioside А_2_-2 from *C. japonica*, as well as their complexes with cholesterol can be considered as potential inhibitors of multidrug resistance of tumor cells. These substances in non-cytotoxic concentrations blocked the activity of the transmembrane transporter P-glycoprotein of mouse Ehrlich carcinoma cells and prevented an efflux of the fluorescent probe Calcein from the cells [83].

Stichoposide C (STC) isolated from *Thelenota anax* induced apoptosis in human leukemia and colorectal cancer cells in a dose-dependent manner and led to the activation of Fas and caspase-8, cleavage of BH3 interacting-domain death agonist (Bid), mitochondrial damage, and activation of caspase-3. Furthermore, STC activated acid sphingomyelinase (SMase) and neutral SMase, that resulted in the generation of ceramide. Specific inhibition of acidic SMase or neutral SMase and siRNA knockdown experiments partially blocked STC-induced apoptosis. Moreover, STC markedly reduced tumor growth of HL-60 xenograft and mouse colon adenocarcinoma CT-26 subcutaneous tumors and increased ceramide generation *in vivo*. It was therefore concluded that STC may have therapeutic relevance for human leukemia and colorectal cancer [84].

## 5. Terpenoids

**Figure 7:**
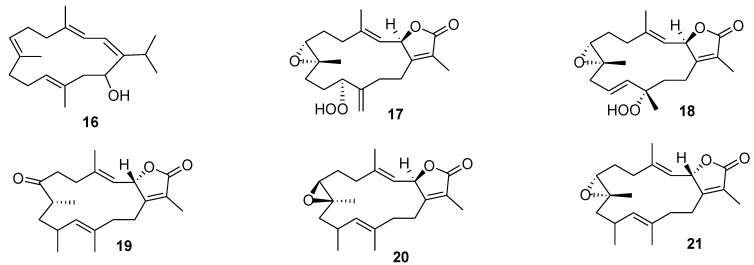
Structures of the compounds **16**–**21**.

Cembrane diterpenoids and their semisynthetic derivatives attracted attention as the potential anticarcinogenic agents. The most intensively investigated cembrane diterpenoid sarcophytol A (Sarc A) (**16**, Figure 7) with the 14-membered ring from the soft coral *Sarcophyton glaucum* inhibited tumor promotion induced by okadaic acid [85], teleocidin [86], and 12-*O*-tetradecanoylphorbol-13-acetate (TPA) [87]. Sarc A inhibited TPA-induced invasion of neutrophils, their levels of myeloperoxidase, and DNA oxidation in the epidermis of sensitive to carcinogenesis (SENCAR) mice exposed to TPA [88]. Inhibition of oxidative stress induced by TPA in human cervix adenocarcinoma HeLa cells led to a 50% decrease in H_2_O_2_ levels when Sarc A was used at a concentration of 75 μM [89]. Nude mice with transplanted human pancreatic cancer cells were fed with a diet containing 0.01% Sarc A. On day 29 after transplantation, tumor volume was significantly smaller in the Sarc A group than in the control group [90]. Sarc A also inhibited methylnitrosourea-induced large bowel-cancer in rats and the development of spontaneous hepatomas in mice [91,92]. 

Recently, a chemical investigation of an ethyl acetate extract of the Red Sea soft coral *Sarcophyton glaucum* has led to the isolation of five cembranoids **17**–**21** (Figure 7). These compounds were found to be the inhibitors of cytochrome P450 1A activity as well as the inducers of glutathione *S*-transferases (GST), quinone reductase (QR), and epoxide hydrolase (mEH), establishing potential modes of action with regards to cancerpreventive activity of these agents [93].

**Figure 8:**
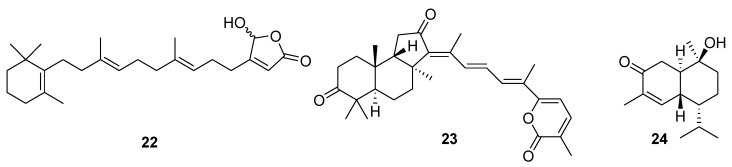
Structures of the compounds **22**–**24**.

Sesterterpenoid luffariellolide (**22**, Figure 8) isolated earlier from the marine sponge *Luffariella* sp. [94], was uncovered as a novel agonist for retinoic acid receptors by inducing co-activator binding to these receptors *in vitro*, further inhibiting cell growth and regulating RAR target genes in various cancer cells [95]. Triterpenoid stellettin A (**23**, Figure 8), obtained from the marine sponge *Geodia japonica*, inhibited the growth of B16 murine melanoma cells by the induction of endoplasmic reticulum stress, abnormal protein glycosylation and autophagy [96]. Cadinane-type sesquiterpene **24** (Figure 8), isolated from the marine-derived fungus *Hypocreales* sp. strain HLS-104, associated with the sponge *Gelliodes carnosa*, showed moderate anti-inflammatory activity in lipopolysaccharide (LPS)-treated RAW264.7 cells with an average maximum inhibition (*E*_max_) value of 10.22% at the concentration of 1 μM [97].

**Figure 9:**
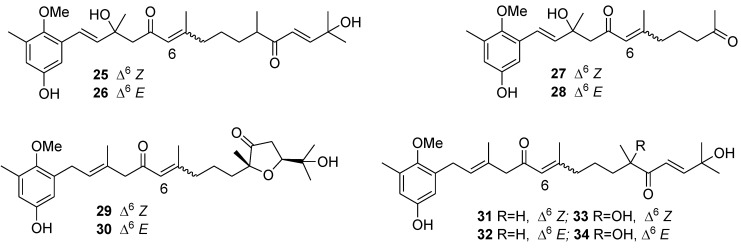
Structures of the compounds **25**–**34**.

A chemical study on the alga *Cystoseira usneoides* has led to the isolation of twelve meroterpenoids. In antioxidant assays, meroterpenes **25**–**32** (Figure 9) exhibited radical-scavenging activity. In anti-inflammatory assays, usneoidone *Z* (**31**) and its 6*E* isomer (**32**) showed significant activities as inhibitors of the production of the proinflammatory cytokine tumor necrosis factor-α (TNF-α) in LPS-stimulated THP-1 human macrophages [98].

**Figure 10:**
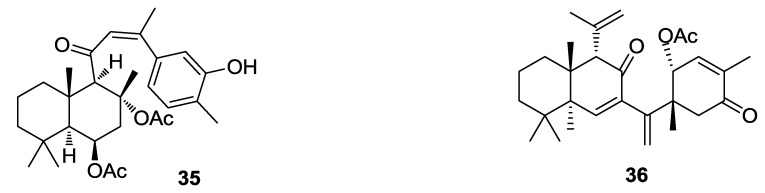
Structures of the compounds **35** and **36**.

Ansellone B (**35**) and phorbasone A acetate (**36**) (Figure 10), isolated from the Korean marine sponge *Phorbas* sp., exhibited potent inhibitory activity on nitric oxide production in RAW 264.7 LPS-activated mouse macrophage cells with IC_50_ values of 4.5 and 2.8 μM, respectively. In particular, ansellone B showed a favorable selectivity index (SI) of 3.8, which is indicative of its inducible isoform nitric oxide synthase (iNOS) inhibitory activity without significant cytotoxicity [99].

**Figure 11:**
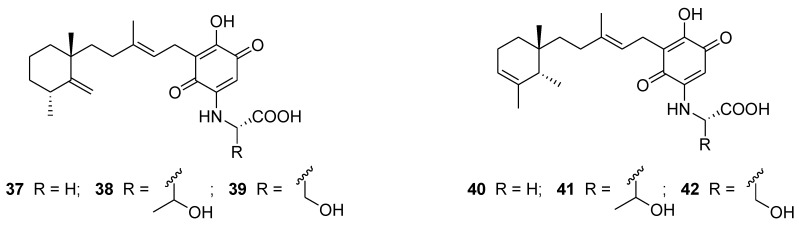
Structures of the compounds **37**–**42**.

Metachromins are a series of sesquiterpenoid quinones with an amino acid residue isolated from Okinawan marine sponges. Metachromins L–Q (**37**–**42**, Figure 11) showed inhibitory activities against receptor tyrosine kinases HER2 with an IC_50_ in the range from 18 to 190 μg/mL [100].

**Figure 12:**
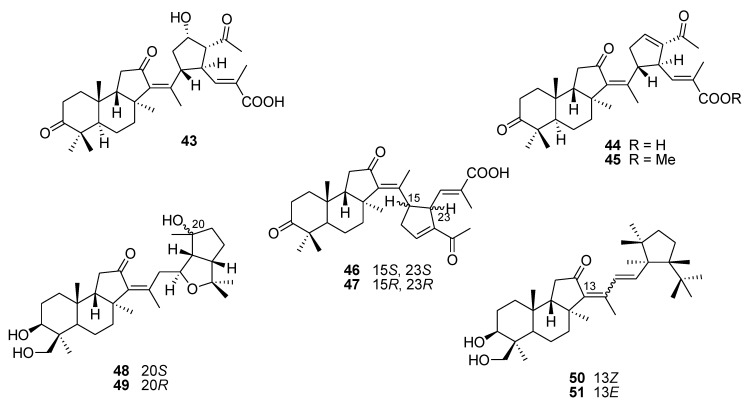
Structures of the compounds **43**–**51**.

Nine unusual isomalabaricane-type triterpenoids with new skeletons in respect to the unprecedented side chains, namely globostelletins J–R (**43**–**51**, Figure 12) were isolated from the marine sponge *Rhabdastrella globostellata*. All compounds were tested for their inhibitory activities against a profile of human tumor-related protein kinases and showed moderate inhibition against the protein kinases ALK (anaplastic lymphoma kinase), FAK (focal adhesion kinase), Aurora-B (serine/threonine kinase), IGF-1R (insulin-like growth factor receptor-1), SRC (proto-oncogene tyrosine-protein kinase), and VEGF-R2 (vascular endothelial growth factor receptor 2) [101].

## 6. Alkaloids

**Figure 13:**
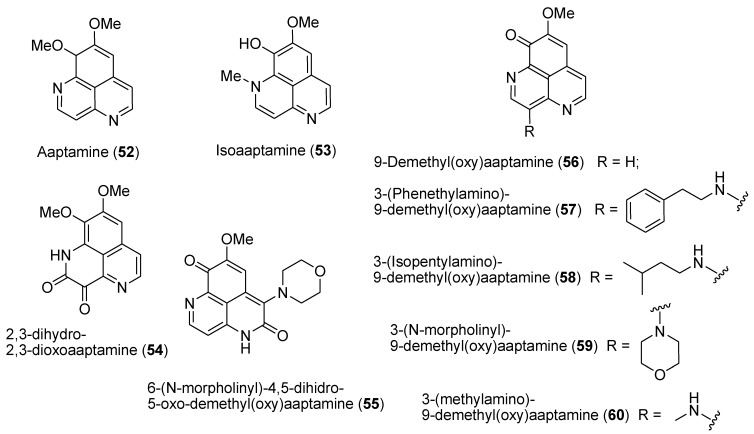
Structures of the compounds **52**–**60**.

The known marine alkaloid aaptamine (**52**, Figure 13) and related compounds **53**–**60** (Figure 13) from the sponge *Aaptos* sp. were shown to inhibit epidermal growth factor (EGF)-induced malignant transformation of mouse epidermal JB6 P^+^Cl41 cells, to possess potent antioxidant properties, and to induce apoptosis in human cancer cells. It was therefore suggested that these agents possess cancer preventive properties [102,103,104]. Recently, the mechanisms of anticancer action of the aaptamine derivatives have become somewhat clearer. Dyshlovoy *et al.* analyzed the effects of aaptamine and its derivatives on the proliferation and protein expression of the pluripotent human embryonal carcinoma cell line NT2. Effects on cell cycle and induction of apoptosis were also analyzed. At lower concentrations, including the IC_50_ of 50 μM, aaptamine treatment resulted in a G2/M arrest, whereas at higher concentrations, induction of apoptosis was observed. Proteomic screening revealed that aaptamine treatment of NT2 cells at the IC_50_ for 48 h resulted in an alteration of 10 proteins. Interestingly, these studies identified the posttranslational hypusine modification of the eukaryotic translation initiation factor 5A-1 (eIF5A) as a prominent target of aaptamine action [105]. It was also shown that aaptamine and its derivatives 9-demethyl(oxy)aaptamine (**56**, Figure 13) and isoaaptamine (**53**, Figure 13) were equally effective as anti-cancer agents in cisplatin-sensitive and -resistant germ cell tumour cells [106]. Again, proteomic profiling was performed, and identified several altered proteins when cisplatin-resistant embryonal carcinoma cells NT2-R were treated with compounds **52**, **53**, and **56** at the corresponding IC_50_.

**Figure 14:**
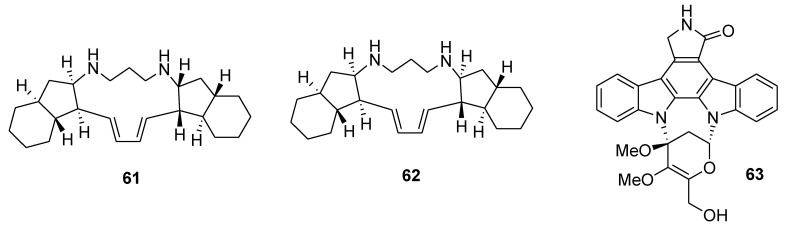
Structures of the compounds **61**–**63**.

Treatment of lymphoma U937 cells with pentacyclic alkaloids, papuamine and haliclonadiamine (**61** and **62**, Figure 14), isolated from an Indonesian marine sponge *Haliclona* sp., resulted in accumulation of cells in the sub-G1 phase, and induced a condensation of chromatin and fragmentation of nucleus [107]. Streptocarbazole A (**63**, Figure 14), isolated from the marine-derived actinomycete strain *Streptomyces* sp., arrested the cell cycle of HeLa cells in the G2/M phase at the noncytotoxic concentration of 10 μM [108].

**Figure 15:**
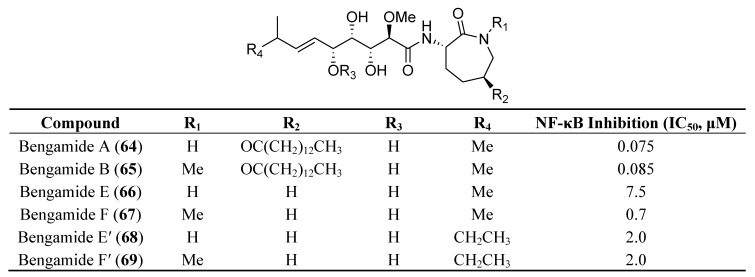
Structures of bengamides **64**–**69** and their effects on the NF-κB activity.

Bengamides **64**–**69** (Figure 15), isolated from two disparate sources, *Myxococcus virescens* (bacterium) and *Jaspis coriacea* (sponge) was shown to be a new class of immune modulators exerting their activity through the inhibition of NF-κB without exerting cytotoxicity in RAW264.7 macrophage immune cells. Western blot and qPCR analysis indicated that bengamides A and B reduce the phosphorylation of nuclear factor of κ light polypeptide gene enhancer in B-cells inhibitor α (IκBα) and the LPS-induced expression of the proinflammatory cytokines/chemokines TNFα, IL-6 and monocyte chemoattractant protein-1 (MCP-1), but do not affect NO production or the expression of iNOS [109].

**Figure 16:**
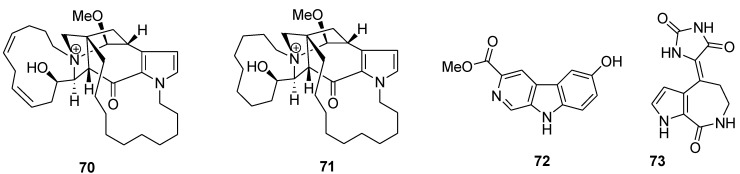
Structures of the compounds **70**–**73**.

New macrocyclic pyrrole alkaloids densanins A and B (**70** and **71**, Figure 16) were isolated from the sponge *Haliclona densaspicula*. The compounds showed relatively potent inhibitory effects on lipopolysaccharide-induced nitric oxide production in BV2 microglial cells, with IC_50_ values of 1.05 and 2.14 μM, respectively [110]. β-Сarboline alkaloid, variabine B (**72**, Figure 16), was isolated from the Indonesian marine sponge *Luffariella variabilis*. The compound inhibited chymotrypsin-like activity of the proteasome and Ubc13 (E2)–Uev1A interaction with IC_50_ values of 4 and 5 μg/mL, respectively [111]. 

The components of the ubiquitin–proteasome system like ubiquitin-specific-processing protease 7 (USP7) have become attractive structures for the development of anticancer agents. USP7, a deubiquitylating enzyme hydrolyzing the isopeptide bond at the *C*-terminus of ubiquitin, is an emerging cancer target. USP7 inhibitors stabilize p53 in cells through degradation of Hdm2 (also known as MDM2), which subsequently results in the suppression of cancer. Spongiacidin C (**73**, Figure 16) isolated from the marine sponge *Stylissa massa* is the first USP7 inhibitor obtained from a natural source. This compound inhibited USP7 with an IC_50_ of 3.8 μM [112].

## 7. Other Low Molecular Weight Marine Natural Compounds

**Figure 17:**
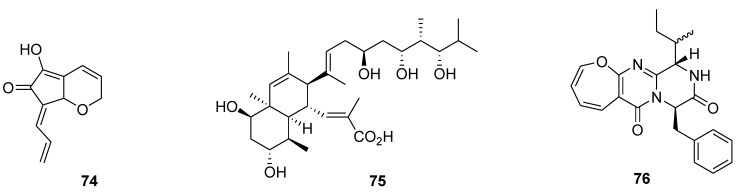
Structures of the compounds **74**–**76**.

Some other marine natural products, isolated from invertebrates, algae and microorganisms showed cancer preventive activities, mainly *in vitro*. For example, C_11_ cyclopentenone, 5-hydroxy-7-prop-2-en-(*E*)-ylidene-7,7*a*-dihydro-2*H*-cyclopenta[*b*]-pyran-6-one (**74**, Figure 17), isolated from a sponge and ascidians, inhibited EGF-induced neoplastic JB6 Cl41 P^+^ cell transformation in soft agar, and induced apoptosis of HL-60 and THP-1 human leukemia cells [113]. 

The highly hydroxylated polyketide nahuoic acid A (**75**, Figure 17), produced in culture by a *Streptomyces* sp. obtained from a marine sediment, is a selective S-adenosylmethionine (SAM) competitive inhibitor of the histone methyltransferase SETD8 *in vitro* [114]. SETD8 is overexpressed in various types of cancer, and aberrant monomethylation by SETD8 may lead to human carcinogenesis [115]. A new oxepin-containing diketopiperazine-type compound protuboxepin A (**76**, Figure 17), binds to α, β-tubulin heterodimers, and accelerates tubulin polymerization *in vitro*, resulting in chromosome misalignment and metaphase arrest which leads to apoptosis in tumor cells [116].

**Figure 18:**
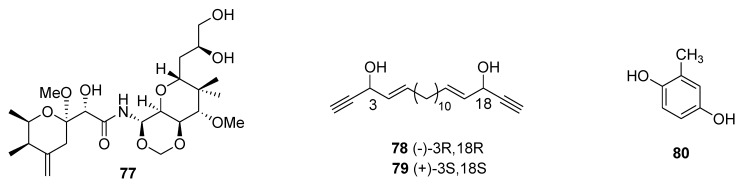
Structures of the compounds **77**–**80**.

Mycalamide A (**77**, Figure 18), isolated from sponges and known as a protein synthesis inhibitor at nanomolar concentrations, inhibits EGF-induced neoplastic transformation and induces caspase-3-dependent apoptosis of mouse JB6 Cl41 P^+^ cells. The compound also inhibits transcriptional activity of oncogenic AP-1 and NF-κB nuclear factors [117]. 

Two enantiomeric C-20 bisacetylenic alcohols **78**; **79** (Figure 18) were isolated from a marine sponge *Callyspongia* sp. as a result of screening of antilymphangiogenic agents from marine invertebrates. Both compounds showed inhibition of the capillary-like tube formation of temperature-sensitive rat lymphatic endothelial from thoracic duct (TR-LE) cells [118]. 

Toluquinol (**80**, Figure 18), a methylhydroquinone produced by a marine fungus, was selected in the course of an unselected screening for new potential inhibitors of angiogenesis. The compound demonstrated antiangiogenic effects both *in vitro* and *in vivo* that were exerted partly by suppression of the VEGF and FGF-induced cytosolic protein kinase Akt activation of endothelial cells [119].

The tricyclic peptides neopetrosiamides A and B, isolated from the marine sponge *Neopetrosia* sp., differ in the stereochemistry of the methionine sulfoxide. They are potential antimetastatic agents that prevent tumour cell invasion by inhibition of both amoeboid and mesenchymal migration pathways [120]. 

**Figure 19:**
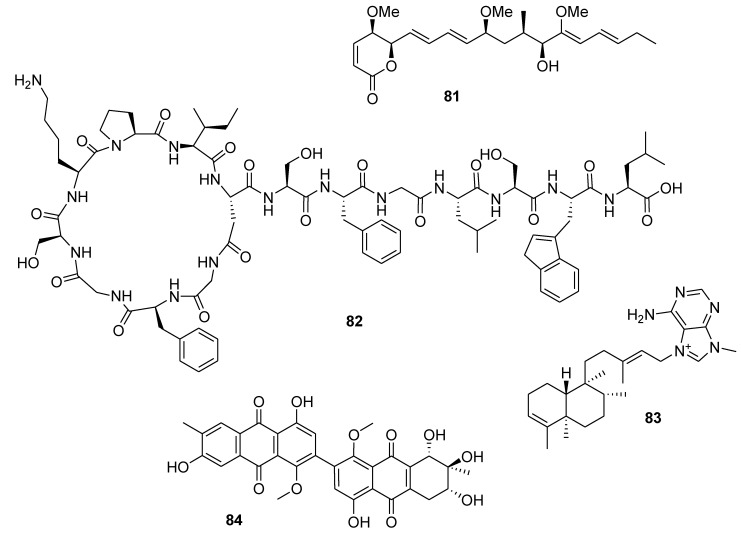
Structures of the compounds **81**–**84**.

Pterocidin (**81**, Figure 19), a linear polyketide with a δ-lactone terminus, was rediscovered from a *Streptomyces* strain of a marine sediment-origin and was found to exhibit potent anti-invasive activity at non-cytotoxic concentrations. The invasion of murine colon 26-L5 carcinoma cells across a matrigel-fibronectin membrane was inhibited by pterocidin at an IC_50_ value of 0.25 μM, whereas the cytotoxicity was not apparent upon treatment with concentrations up to 7 μM [121]. 

Sungsanpin (**82**, Figure 19) from a *Streptomyces* species, isolated from deep-sea sediment, displayed inhibitory activity in a cell invasion assay using the human lung cancer cell line A549 [122]. Agelasine B (**83**, Figure 19), a compound purified from the marine sponge *Agelas clathrodes*, induced fragmentation of DNA, reduced the expression of Bcl-2, and was able to activate caspase 8 in MCF-7 human breast cancer cells [123]. Alterporriol L (**84**, Figure 19), a new bianthraquinone derivative, was isolated from the marine fungus *Alternaria* sp. The reactive oxygen species, mitochondrial membrane potential, and cytosolic free calcium level in MCF-7 breast cancer cells were changed after treatment with alterporriol L, suggesting that alterporriol L plays a vital role in cancer cells through destroying the mitochondrial potential and inducing apoptosis [124].

**Figure 20:**
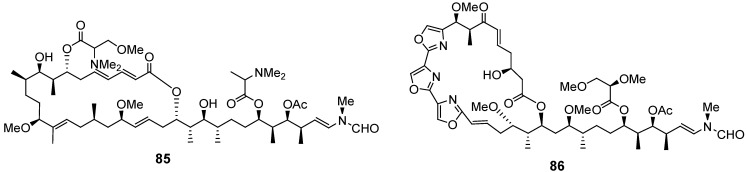
Structures of the compounds **85** and **86**.

Marine macrolides aplyronine A and mycalolide B (**85** and **86**, Figure 20) were shown to induce apoptosis in human leukemia HL60 cells and human epithelial carcinoma HeLa S3 cells [125].

**Figure 21:**
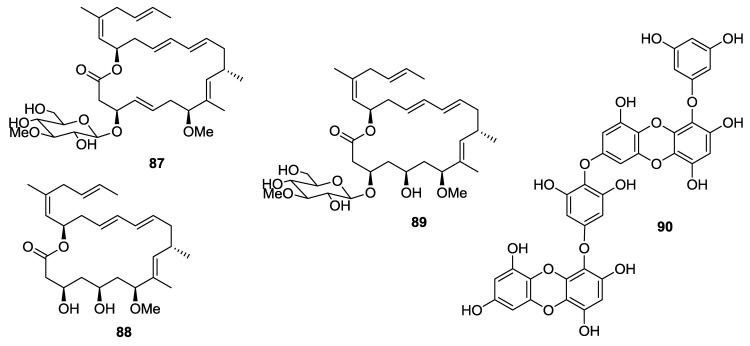
Structures of the compounds **87**–**90**.

Biselyngbyaside (**87**), biselyngbyolide A (**88**), and biselyngbyaside B (**89**) (Figure 21), isolated from the marine cyanobacterium *Lyngbya* sp., induced apoptosis and increased cytosolic Ca^2+^ concentration in human cancer HeLa S_3_ and HL60 cells [126]. 

Dieckol (**90**, Figure 21), a nutrient polyphenol compound from the brown alga *Ecklonia cava,* inhibited migration and invasion of human fibrosarcoma HT1080 cells by scavenging intracellular reactive oxygen species (ROS). Dieckol treatment also decreases complex formation of focal adhesion kinase (FAK)-proto-oncogene tyrosine-protein kinase Src-p130 Crk-associated substrate (p130Cas) and expression of MMP2, 9, and 13 [127].

**Figure 22:**
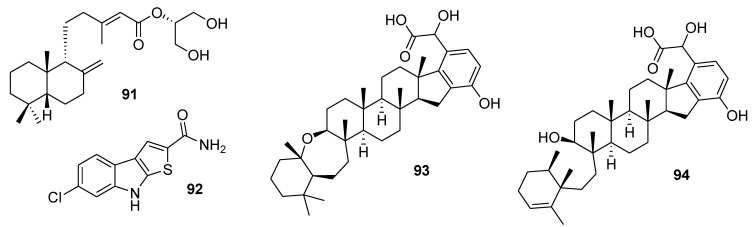
Structures of the compounds **91**–**94**.

Palmadorin M (**91**, Figure 22), isolated from the Antarctic nudibranch *Austrodoris kerguelenensis,* inhibited Janus kinase 2 (Jak2), signal transducer and activator of transcription 5 (STAT5), and extracellular signal-regulated kinase 1/2 (Erk1/2) activation in human erythroleukemia HEL cells and caused apoptosis at a concentration of 5 μM [128]. 

Thienodolin (**92**, Figure 22), isolated from a *Streptomyces* sp. derived from Chilean marine sediment, inhibited nitric oxide production in LPS-stimulated RAW 264.7 cells (IC_50_ = 17.2 ± 1.2 μM). At both the mRNA and protein levels, inducible nitric oxide synthase (iNOS) was suppressed in a dose dependent manner. The compound blocked the degradation of IκBα, resulting in an inhibition of NF-κB p65 nuclear translocation, and inhibited the phosphorylation of the signal transducer and activator of transcription (STAT1) at Tyr701 [129].

Two new merohexaprenoids, halicloic acids A and B (**93** and **94**, Figure 22), have been isolated from the marine sponge *Haliclona (Halichoclona)* sp., collected in the Philippine waters. The compounds showed inhibition of indoleamine 2,3-dioxygenase (IDO) at 10 and 11 μM, respectively [130].

**Figure 23:**
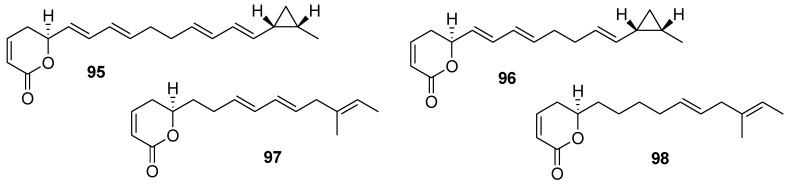
Structures of the compounds **95**–**98**.

Polyketides coibacins **95**–**98** (Figure 23), isolated from a Panamanian marine cyanobacterium cf. *Oscillatoria* sp., were tested for anti-inflammatory activity in a cell-based nitric oxide (NO) inhibition assay. In this assay, coibacin B (**96**) was determined to be the most active of these natural compounds. Coibacin A (**95**) significantly reduced gene transcription of four cytokines (TNF-R, IL-6, IL-1β, and iNOS), with especially notable effects on IL-1β and iNOS, at a concentration of 10 μg/mL. Using ELISA, changes in protein expression for some of these inflammatory cytokines were measured in murine RAW264.7 cells stimulated with lipopolysaccharide (LPS) in the absence or presence of the coibacins. Coibacin A (**95**) at 10 μg/mL was found to significantly reduce TNF-R and IL-6 secretion. Coibacins B-D (**96**–**98**) also affected protein expression of TNF-R and IL-6, albeit to a lesser extend [131].

**Figure 24:**
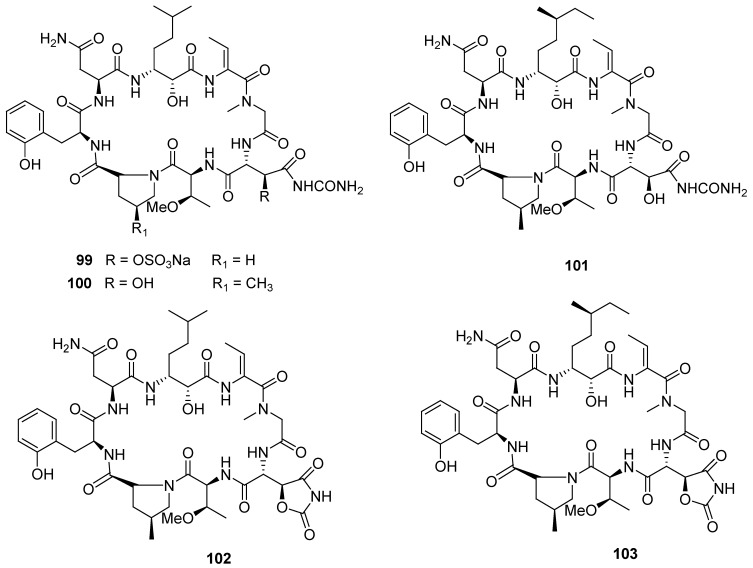
Structures of the compounds **99**–**103**.

Five new cyclopeptides, perthamides G-K (**99**–**103**, Figure 24), were isolated from the polar extract of the marine sponge *Theonella swinhoei*. Pharmacological analysis demonstrated that these natural cyclopeptides are endowed with anti-inflammatory potential, as assessed by their ability to reduce carrageenan-induced mouse paw oedema [132].

**Figure 25:**
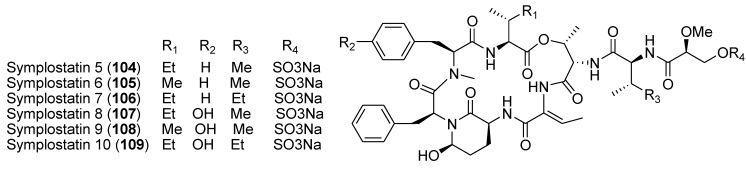
Structures of the compounds **104**–**109**.

New elastase inhibitors symplostatins 5–9 (**104**–**109**, Figure 25) were isolated from red cyanobacterium collected from Cetti Bay, Guam. Symplostatin 5 (**104)** was shown to attenuate the downstream cellular effects of elastase in an epithelial lung airway model system, alleviating clinical hallmarks of chronic pulmonary diseases such as cell death, cell detachment, and inflammation. This compound attenuated the effects of elastase on receptor activation, proteolytic processing of the inter-cellular adhesion molecule-1 (ICAM-1), NF-κB activation, and transcriptomic changes, including the expression of pro-inflammatory cytokines IL1A, IL1B, and IL8 [133].

**Figure 26:**
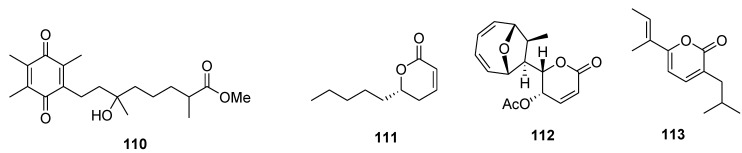
Structures of the compounds **110**–**113**.

Flexibilisquinone (**110**, Figure 26), isolated from the cultured soft coral *Sinularia flexibilis*, originally distributed in the waters of Taiwan, was found to inhibit the accumulation of the pro-inflammatory iNOS and cyclooxygenase-2 (COX-2) proteins of LPS-stimulated RAW264.7 macrophage cells [134]. Compound **111** (Figure 26), isolated from the marine-derived fungus *Hypocreales* sp. strain HLS-104, isolated from a sponge *Gelliodes carnosa*, was effective against the nitric oxide (NO) production in lipopolysaccharide (LPS)-treated RAW264.7 cells and showed moderate inhibition with *E*_max_ values of 26.5% at a concentration of 1 μM [97]. 

Mycoepoxydiene (MED) (**112**, Figure 26) is a polyketide isolated from the marine fungal strain *Diaporthe* sp. HLY-1. MED induced DNA damage through the production of reactive oxygen species (ROS), which resulted in the phosphorylation of H2A histone family memeber X (H2AX) and the activation of the Ataxia telangiectasia mutated kinase (ATM) and p53 signaling pathways. In addition, MED increased the accumulation of IkBα and enhanced the association between IκB kinase γ (IKKγ) and heat shock protein 27 (Hsp27) via the activation of Hsp27, which eventually resulted in the inhibition of TNF-α-induced NF-κB transactivation [135]. 

Nocapyrone H (**113**, Figure 26) isolated from the marine actinomycete *Nocardiopsis* sp. KMF-001 reduced the pro-inflammatory factors such as nitric oxide (NO), prostaglandin E-2 (PGE(2)) and interleukin-1 β (IL-1 β). Moreover, nocapyrone H showed a 5.8% stronger inhibitory effect on NO production than chrysin at a concentration of 10 μM in lipopolysaccharide (LPS)-stimulated BV-2 microglial cells [136].

**Figure 27:**
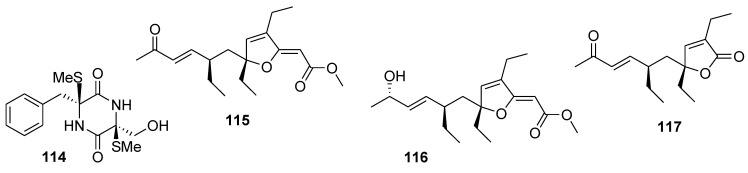
Structures of the compounds **114**–**117**.

Bis-*N*-norgliovictin (**114**, Figure 27), a small molecule, isolated during the screening of natural products against inflammation from the culture broth of a marine derived fungus named S3-1-c, significantly inhibited LPS (ligand of toll-like receptor 4 (TLR4))-induced TNF-α production in RAW264.7 cells. In this cell line and in mouse peritoneal macrophages, bis-*N*-norgliovictin inhibited LPS-induced production of TNF-α, IL-6, interferon-β (IFN-β) and monocyte chemoattractant protein (MCP-1) in a dose-dependent manner, without suppressing cell viability. The anti-inflammatory effect was attributed to the down-regulation of the TLR4-triggered myeloid differentiation primary response protein 88 (MyD88) and TIR-containing adapter inducing interferon-β (TRIF) signaling pathways, including p38 and c-Jun *N*-terminal kinase (JNK) of mitogen-activated protein kinases (MAPKs), NF-κB, and interferon regulatory factor 3 (IRF3) cascades. Importantly, bis-*N*-norgliovictin also protected mice against LPS-induced endotoxic shock [137]. 

Biochemical characterization of a library of 13 oxygenated polyketides isolated from the marine sponge *Plakinastrella mamillaris* led to the discovery of gracilioethers B and C, and plakilactone C (**115**, **116**, and **117**, Figure 27) as selective PPARγ ligands in transactivation assays [138]. PPAR ligands activated PPAR signaling and exerted cancer-preventive and cytotoxic effects *in vitro* and/or *in vivo* [31].

**Figure 28:**
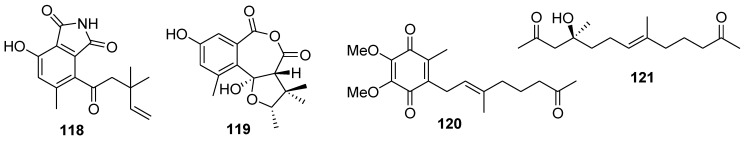
Structures of the compounds **118**–**121**.

Isoindole pseudoalkaloid conioimide (**118**, Figure 28) and the polyketide cereoanhydride (**119**, Figure 28) were isolated from the fungus *Coniothyrium cereale* isolated from the Baltic Sea alga *Enteromorpha* sp. Conioimide has prominent and selective inhibitory activity towards the protease human leukocyte elastase (HLE), an enzyme involved in many inflammatory diseases, with an IC_50_ value of 0.2 μg/mL, whereas cereoanhydride showed weaker inhibition (IC_50_ = 16 μg/mL) [139]. 

A ubiquinone derivative, pseudoalteromone A (**120**, Figure 28), possessing a 9C nor-monoterpenoid moiety, and a 15C compound, pseudoalteromone B (**121**, Figure 28), were obtained from the marine bacterium *Pseudoalteromonas* sp. CGH2XX, originally isolated from a cultured-type octocoral *Lobophytum crassum*. Pseudoalteromones A and B exhibited anti-inflammatory activity through inhibitory effects (inhibition rates 45.1% and 20.7%, correspondingly) on the release of elastase by human neutrophils at a concentration of 10 μg/mL [140,141].

**Figure 29:**
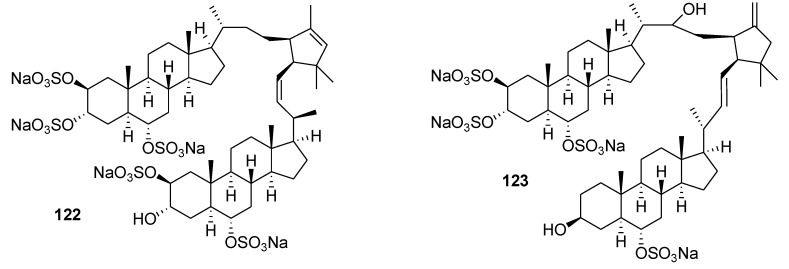
Structures of the compounds **122** and **123**.

Two dimeric sterols, manadosterols A and B (**122** and **123**, Figure 29), were isolated from the marine sponge *Lissodendryx fibrosa* collected in Indonesia. The compounds inhibited the Ubc13-Uev1A interaction with IC_50_ values of 0.09 and 0.13 μM, respectively [142] They are the second and third natural compounds showing inhibitory activities against the Ubc13–Uev1A interaction and are more potent than leucettamol A (IC_50_, 106 μM), the first such inhibitor, isolated from another marine sponge [44].

**Figure 30:**
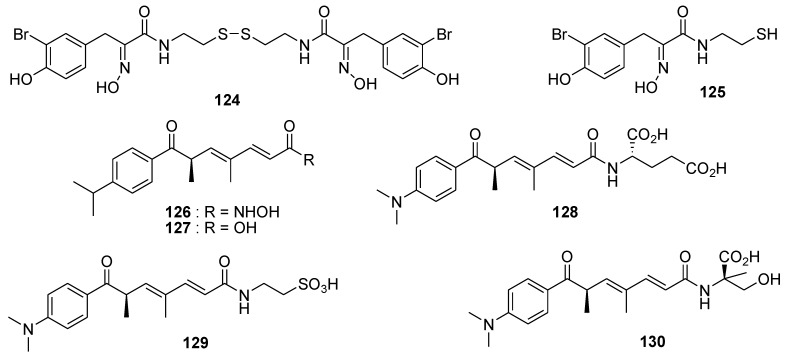
Structures of the compounds **124**–**130**.

The symmetrical disulfide psammaplin A (**124**, Figure 30) was isolated from the marine sponge *Pseudoceratina* sp. and could be interesting for treatment approaches targeting epigenetic alterations, since it showed potent inhibition of histone deacetylases (HDAC, IC_50_ 4.2 ± 2.4 nM) [143]. The psammaplin-derived thiol (**125**, Figure 30) exhibited potent activity against histone deacetylases in a low nanomolar range, but showed low cytotoxicity [144]. Five HDAC1 inhibitors, trichostatin A (**126**, Figure 30) and its analogues trichostatic acid, JBIR-109, JBIR-110, and JBIR-111 (**127**–**130**, Figure 30) were isolated from the culture of the marine sponge-derived *Streptomyces* sp. strain RM72. The IC_50_ values against HDAC1 of **126**–**130** were 0.012, 73, 48, 74, and 57 μM, respectively [145].

**Figure 31:**
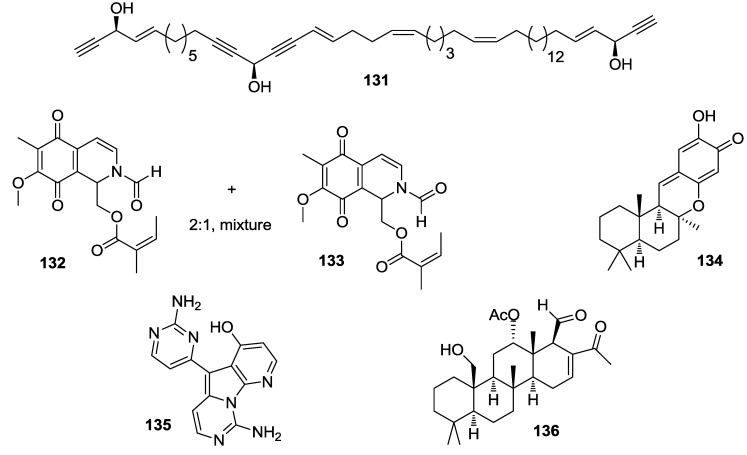
Structures of the compounds **131**–**136**.

Ten extracts from various marine sponges were identified as containing inhibitors of the transcription factor HIF-2α which has been shown to play a distinct role in tumorigenesis. Chemical exploration of these sponge extracts led to isolation of seven specific HIF inhibitors, compounds **131**–**136** (Figure 31) and haliclonadiamine (**62**) [146].

**Figure 32:**
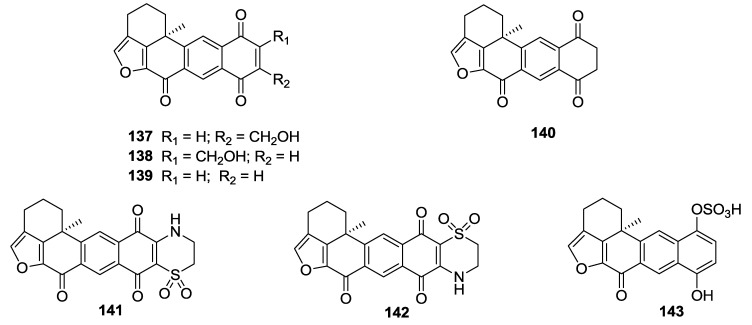
Structures of the compounds **137**–**143**.

The organic extract of a marine sponge, *Petrosia alfiani*, selectively inhibited iron chelator-induced hypoxia-inducible factor-1 (HIF-1) activation in a human breast tumor T47D cell-based reporter assay. Bioassay-guided fractionation yielded seven xestoquinones **137**–**143** (Figure 32). Among them, compounds **141** and **142**, which possess a 3,4-dihydro-2*H*-1,4-thiazine-1,1-dioxide moiety, potently and selectively inhibited HIF-1 activation in T47D cells, each with an IC_50_ value of 0.2 μM, whereas other compounds showed IC_50_ in the range of 1.2 to 30 μM [147].

**Figure 33:**
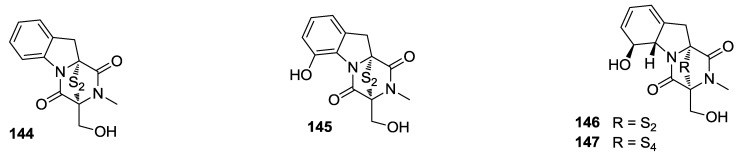
Structures of the compounds **144**–**147**.

Gliotoxin-related compounds **144**–**147** (Figure 33), containing a disulfide or tetrasulfide bond, were isolated from the fungus *Penicillium* sp. strain JMF034, obtained from deep sea sediments of Suruga Bay, Japan. They showed inhibitory activity against histone methyltransferase (HMT) G9a [148].

**Figure 34:**
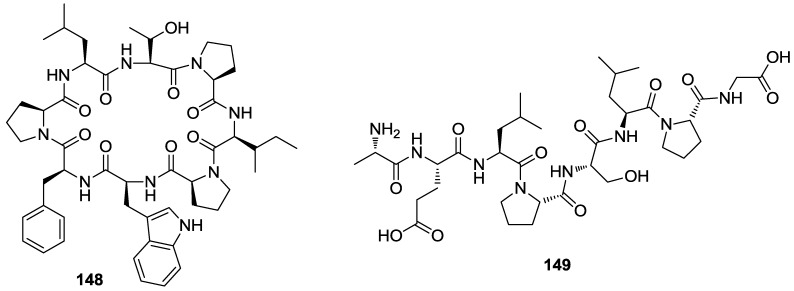
Structures of the compounds **148** and **149**.

A new proline-rich cyclic octapeptide named stylissamide X **148** (Figure 34) was isolated from an Indonesian marine sponge *Stylissa* sp. as an inhibitor of cell migration using a wound-healing assay. Compound **148** showed inhibitory activity against migration of HeLa cells in the concentration range of 0.1–10 μM in both a wound-healing assay and a chemotaxicell chamber assay, while cell viability was maintained at more than 75% at even the highest concentration of 10 μM [149]. 

The oligopeptide **149** (Figure 34) was isolated from the digests of abalone *Haliotis discus hannai* intestine. This marine gastropod is an important fishery and food industrial resource that is massively maricultured in Asia, Africa, Australia, and America. The purified abalone oligopeptide **149** (AOP) exhibited a specific inhibitory effect against MMP-2/-9 activity and attenuated protein expression of p50 and p65 in human fibrosarcoma (HT1080) cells via the nuclear factor-κB (NF-κB) pathway. This data suggest that AOP may possess therapeutic and preventive potential for the treatment of MMPs-related disorders such as angiogenesis and metastasis formation [150].

**Figure 35:**
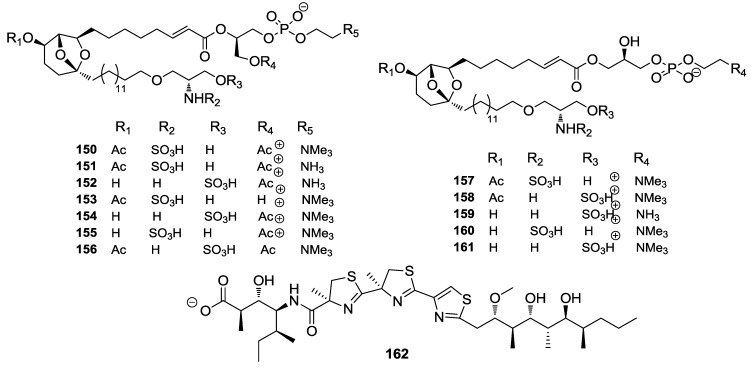
Structures of the compounds **150**–**162**.

Targeting Mdm2/Hdm2 is a promising way to reactivate p53, inducing apoptosis in transformed human cells. New sulfonated serinol derivatives, siladenoserinols A–L (**150**–**161**, Figure 35) were isolated from a tunicate of the family Didemnidae as inhibitors of p53-Hdm2 interaction. The compounds inhibited p53-Hdm2 interaction with IC_50_ values ranging from 2.0 to 55 μM. Among them, siladenoserinol A and B (**150**, **151**) exhibited the strongest inhibition with an IC_50_ value of 2.0 μM [151]. Reactivation of p53 via this approach is also considered a potential way to cancer-prevention, although this needs further study.

Bioassay-guided fractionation of two cyanobacterial extracts from Papua New Guinea has yielded hoiamide D (**162**, Figure 35), a polyketide synthase (PKS)/non-ribosomal peptide synthetase (NRPS)-derived natural product that features two consecutive thiazolines and a thiazole, as well as a modified isoleucine residue. Hoiamide D (**162**) displayed inhibitory activity against p53/Mdm2 interaction (EC_50_ = 4.5 μM) [152].

**Figure 36:**
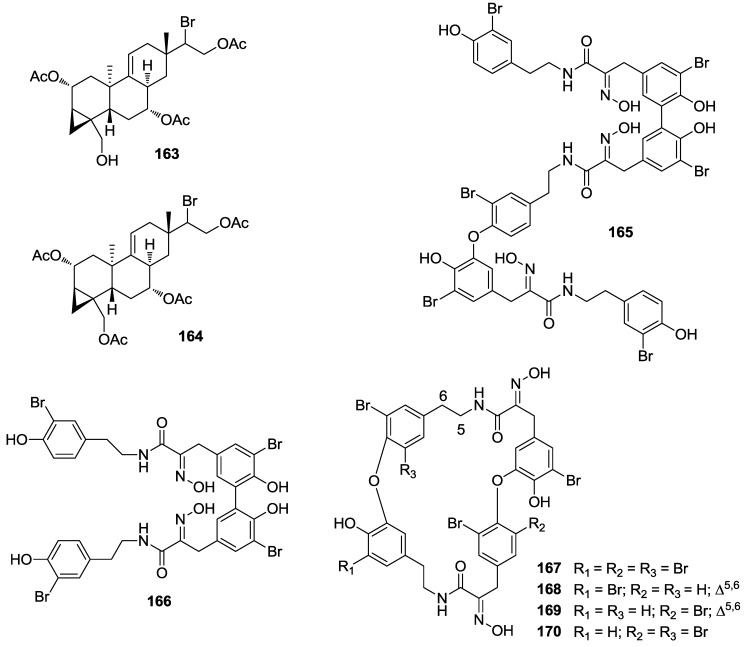
Structures of the compounds **163**–**170**.

Two rare bromoditerpenes, parguerenes I and II (**163**, **164**, Figure 36), were isolated as P-gp inhibitors from a southern Australian collection of the red alga *Laurencia filiformis*. It was determined that the parguerenes were non-cytotoxic, dose-dependent inhibitors of P-gp mediated drug efflux, by modifying the extracellular antibody binding epitope of P-gp in a manner that differs markedly from that of the other known P-gp inhibitors verapamil and cyclosporine A [153]. Their cancer preventive activities, however, have not been studied yet.

Naturally occurring trimeric hemibastadin congeners, sesquibastadin 1 (**165**, Figure 36), and bastadins 3, 6, 7, 11, and 16 (**166**–**170**, Figure 36) were isolated from the marine sponge *Ianthella basta*, collected in Indonesia. The compounds showed inhibition of several protein kinases with IC_50_ values between 0.1 and 10.2 μM [154]. They may well be interesting for their potential anticancer and chemo-preventive properties.

## 8. Conclusions

Many secondary metabolites, isolated from marine organisms in recent years, were shown to be potential anticarcinogenic and chemo-preventive agents using mainly *in vitro* and sometimes *in vivo* experiments. Among these agents are compounds with such different activities as inhibition of transformation of normal cells into cancer cells, abrogation of tumor cell growth and formation of microtumors, and induction of apoptosis. Some compounds showing mainly antioxidative, immunostimulatory, and anti-inflammatory activity also may be promising agents for cancer prevention. Notably, the availability of ever more precise research tools allows the dissection of the molecular mode of action of many natural compounds, which will lead to a more differentiated therapeutic use of these agents in the era of “targeted therapy”. Numerous reports cited in this review clearly indicate that marine organisms are an irreplaceable source of bioactive and often low toxic compounds, which may play an important role in prevention and inhibition of cancer development in humans in the near future. Particularly, natural products isolated from edible species seem an attractive source of cancer-preventive agents. Interdisciplinary studies on marine natural products and close cooperation of bioorganic chemists with the molecular biologists, pharmacologists, and clinicians should help to find new and effective ways of cancer prevention.
